# Imaging lifespan brain structural growth: From region, to connectome, to gradient

**DOI:** 10.1371/journal.pbio.3002669

**Published:** 2024-06-21

**Authors:** Xing Qian, Juan Helen Zhou

**Affiliations:** 1 Centre for Sleep and Cognition & Centre for Translational Magnetic Resonance Research, Yong Loo Lin School of Medicine, National University of Singapore, Singapore; 2 Department of Medicine & Human Potential Translational Research Program, Yong Loo Lin School of Medicine, National University of Singapore, Singapore; 3 Integrative Sciences and Engineering Programme, NUS Graduate School, National University of Singapore, Singapore; 4 Department of Electrical and Computer Engineering, National University of Singapore, Singapore

## Abstract

During development and aging the brain undergoes intricate structural changes. This Primer explores the implications of a new study in PLOS Biology, which provides a detailed characterization of how the brain’s morphometric organization changes throughout the human lifespan to support cognition.

## Teaser

Throughout human life, the brain undergoes intricate structural changes that support cognition. A study in *PLOS Biology* introduces new avenues for depicting the trajectory of the brain morphometric connectome and its underlying genetic and molecular mechanisms.

Growth charts quantify age-related changes in body measurements and are used to monitor the growth and development in children from birth to school age. A recent seminal work modernized this concept for building normative charts of brain morphometric measures derived from structural magnetic resonance imaging (MRI), i.e., brain charts over the entire life-course [1]. Brain charts are an essential step towards robust quantification of individual variation benchmarked to normative trajectories, paving the way for precision medicine in brain disorders. In this Primer, we discuss a new study in this issue by Li and colleagues [2] and highlight perspectives and prospects for establishing brain structural growth charts from region, to connectome, to gradient, across the lifespan.

Increasing efforts have been invested in mapping the large-scale network architecture of anatomically connected regions in the human brain, i.e., brain connectome charts across the lifespan. Structural covariance networks (SCNs) show convergent patterns with known brain functional networks (via resting functional MRI), capturing spatially and temporally coordinated mechanisms of maturation or degeneration in brain regions [3]. Instead of estimating SCNs at the group level using a single morphometric feature, Seidlitz and colleagues previously invented a novel technique to map individual-level morphometric similarity networks (MSNs), based on the interregional similarity of multiple morphometric parameters [4]. Notably, MSN topology captures known cortical cytoarchitecture, related gene expression, between-subject variation in human intelligence, and disease-related changes.

In contrast to network or parcellation analysis, which emphasizes universality within networks and heterogeneity among networks or parcels, the connectome gradient technique allows researchers to explore the hierarchical architecture in the brain connectome. It rearranges the neural elements into a collection of gradients according to the similarity of their connectivity patterns using dimension reduction procedures. Converging evidence demonstrates that the functional connectome of the adult human brain is organized along two core connectivity gradients, including the principal gradient from the primary sensorimotor and visual cortex to the transmodal regions, and the secondary gradient separating sensorimotor areas and the visual cortex [5].

Li and colleagues [2] projected individual cortical MSNs into 3D gradient space and calculated within- and between-network dispersions (i.e., Euclidean distances) within this space, which capture multidimensional differences in cortical MSN organization. By leveraging structural MRI scans from 1,790 individuals aged 8 to 89, they examined the human lifespan trajectory of the cortical dispersion of MSNs, which is an important step forward in delineating growth charts of the brain connectome.

The principal organization of the brain connectome extending from the unimodal sensory cortex to the transmodal association cortex supports the propagation of hierarchical information between unimodal networks (for instance, immediate perception) and transmodal networks (for instance, executive functions, socioemotional processing, and mentalizing abilities). Such brain network architecture follows complex lifespan trajectories, aligning with milestones in cognitive and behavioral capabilities [6]. For instance, Pines and colleagues observed that the developmental patterns in youth differentially unfold along the unimodal–transmodal hierarchy: Unimodal sensorimotor networks became more integrated with age, while transmodal association networks became more segregated with age, which related to the emergence of executive function [7].

The new research by Li and colleagues [2] reinforces this dissociable maturation pattern: The primary motor class showed decreased within-network dispersion, while the association classes showed increased within-network dispersion from late childhood to adolescence. Importantly, the authors found that the dispersions of primary motor and association cortices jointly mediated the relationships between age and cognitive flexibility during late childhood to adolescence, while such effect was missing in young, middle, and late adulthood. This finding underscores the critical role of brain connectome maturation in adolescence. Nonetheless, the complex and nonlinear patterns of morphometric reorganization during development (or aging), and their associations with cognitive functions, require finer delineation using large sample sizes with balanced distribution across age groups.

The extensive maturation and reorganization of the brain during development and aging are influenced by a combination of genetically determined biological processes and environmental interactions [8]. Emerging evidence supports a link between microscale properties, such as transcription profiles, cytoarchitecture, neurotransmitter receptor densities, and laminar differentiation, and the macroscopic organization of brain networks [9]. These local attributes influence the broadcasting and integration of signal traffic within neuronal populations, potentially shaping the structural and functional organization of the human connectome. However, molecular contributions to age-related brain network reorganization have been relatively understudied. To address this gap, Li and colleagues [2] revealed that age-related changes in global dispersion unfolded along patterns of molecular brain organization, including the density distributions of acetylcholine receptor and, possibly, glutamate and dopamine neurotransmitter receptors. Additionally, they decoded the global dispersions with postmortem gene expression maps. Although more validation using tissue-specific gene-expression analysis is needed, the current work provides novel insights into the genetic and molecular mechanisms underpinning age-related brain connectome reorganization.

**Fig 1:**
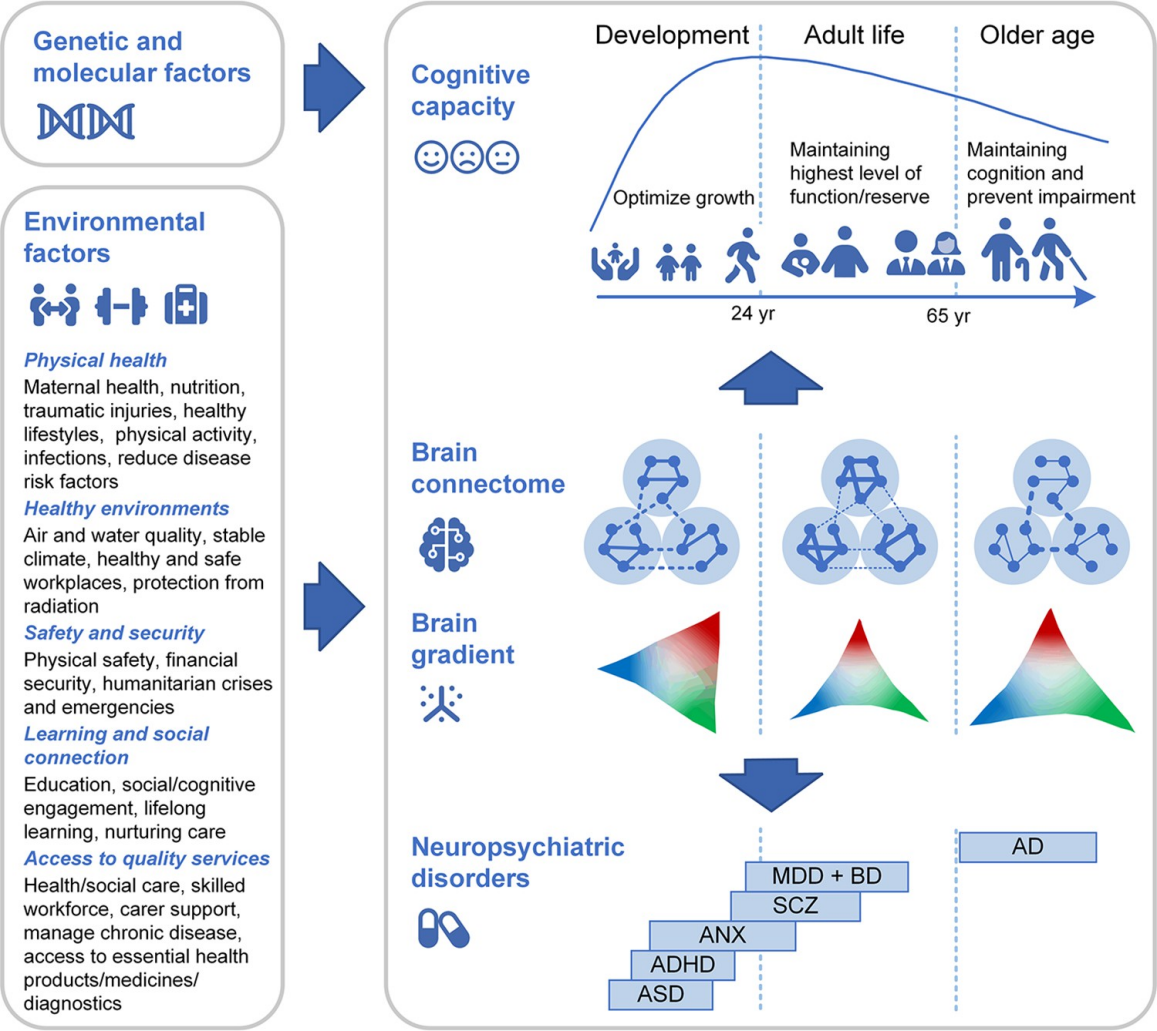
Genetics, molecular factors, and environmental interactions affect lifespan brain connectome charts, underpinning cognitive capacity and neuropsychiatric disorders. Right top: Schematic representation of the trajectory of cognitive development over the life-course [6]. Right middle: Schematic illustration of the lifespan trajectory of brain connectome and gradient [11]. Brain modules become more segregated during youth. The older adult brain is less modular, more integrated, and less efficient compared to young adults. Right bottom: A graphical summary of the age range in which major psychiatric conditions are generally diagnosed as derived from literature [1]. Left: The life-course brain network trajectory and its effect on cognition and mental health may vary according to factors including genetics and molecular biology, and the interaction of lifestyle, environment, education, and health [12]. AD, Alzheimer’s disease; ADHD, attention deficit hyperactivity disorder; ASD, autism spectrum disorder (including high-risk individuals with confirmed diagnosis at a later age); ANX, anxiety or phobic disorders; BD, bipolar disorder; MDD, major depressive disorder; RMR, resting metabolic rate; SCZ, schizophrenia.

Looking ahead, we can identify several directions beyond the current research (Fig 1). First, while the cross-sectional design used in this work can reveal new insights, longitudinal designs should be employed to characterize possibly nonlinear within-subject trajectories and interactions with other factors over time. Second, it is crucial to develop robust and biologically plausible approaches for morphometric similarity mapping at the individual level. Together with the commonly validated brain network mapping methods, including white matter fiber tractography and functional network mapping, multimodal brain connectome charts provide new opportunities to reveal comprehensive principles of cortical network organization throughout normative processes of brain development and aging. Third, there are still gaps in our understanding of how environmental factors interact with genetic and molecular contributions to brain network reorganization. The social environment, such as socioeconomic status and family support, has a profound impact on brain development, which then shapes risk of and resilience to mental health difficulties [10]. Moreover, maintaining a socially active lifestyle in later life may enhance cognitive reserve [6]. It is therefore crucial to examine the precise influence of genetic and environmental factors to promote a resilient brain at the region, connectome, and gradient level. Lastly, while Li and colleagues [2] focused on cognitive flexibility, future studies should examine how brain structural measures across various levels relate to emotion, memory, personality, and mental health.

By harnessing these collective efforts, we anticipate that brain structural connectome charts will provide a practical and insightful understanding of how human cortical networks contribute to individual variations in psychological functions, with profound implications for early prediction and intervention in neuropsychiatric disorders.

## Abbreviations

MRI: magnetic resonance imaging
MSN: morphometric similarity network
SCN: structural covariance network
